# Predicting Impaired Extinction of Traumatic Memory and Elevated Startle

**DOI:** 10.1371/journal.pone.0019760

**Published:** 2011-05-18

**Authors:** Rebecca Nalloor, Kristopher Bunting, Almira Vazdarjanova

**Affiliations:** Brain and Behavior Discovery Institute and Department of Neurology, Georgia Health Sciences University, Augusta, Georgia, United States of America; University of Sydney, Australia

## Abstract

**Background:**

Emotionally traumatic experiences can lead to debilitating anxiety disorders, such as phobias and Post-Traumatic Stress Disorder (PTSD). Exposure to such experiences, however, is not sufficient to induce pathology, as only up to one quarter of people exposed to such events develop PTSD. These statistics, combined with findings that smaller hippocampal size prior to the trauma is associated with higher risk of developing PTSD, suggest that there are pre-disposing factors for such pathology. Because prospective studies in humans are limited and costly, investigating such pre-dispositions, and thus advancing understanding of the genesis of such pathologies, requires the use of animal models where predispositions are identified *before* the emotional trauma. Most existing animal models are retrospective: they classify subjects as those with or without a PTSD-like phenotype long after experiencing a traumatic event. Attempts to create prospective animal models have been largely unsuccessful.

**Methodology/Principal Findings:**

Here we report that individual predispositions to a PTSD-like phenotype, consisting of impaired rate and magnitude of extinction of an emotionally traumatic event coupled with long-lasting elevation of acoustic startle responses, can be revealed following exposure to a mild stressor, but before experiencing emotional trauma. We compare, in rats, the utility of several classification criteria and report that a combination of criteria based on acoustic startle responses and behavior in an anxiogenic environment is a reliable predictor of a PTSD-like phenotype.

**Conclusions/Significance:**

There are individual predispositions to developing impaired extinction and elevated acoustic startle that can be identified after exposure to a mildly stressful event, which by itself does not induce such a behavioral phenotype. The model presented here is a valuable tool for studying the etiology and pathophysiology of anxiety disorders and provides a platform for testing behavioral and pharmacological interventions that can reduce the probability of developing pathologic behaviors associated with such disorders.

## Introduction

Experiencing emotional trauma, with or without physical trauma, leads to debilitating pathological anxiety and impairment in social and cognitive function, called Post Traumatic Stress Disorder in almost one quarter of exposed people [1], [2]. Current PTSD research focuses on finding treatments that allow patients to successfully cope with a traumatic event in the immediate aftermath of that event [3], [4]. However, the fact that a traumatic incident does not affect all subjects equally suggests that there are individual risk factors which predispose them to developing PTSD. The availability of pre-trauma classification can be very helpful in correctly identifying pharmacological and behavioral treatments that are likely to benefit susceptible populations. Recognizing such benefits, studies in humans are underway [3], [5].

Existing animal models have contributed greatly to the understanding of the disease symptoms that develop after emotional trauma and the possible treatment of these symptoms [6]–[18]. However, the investigation of memory processes occurring during or shortly after the traumatic event is not currently possible. Here we present a different model that will allow such investigations and can serve as a platform for testing the effectiveness of pre-trauma and peri-trauma interventions.

Hallmarks of trauma-based anxiety disorders, such as PTSD, are exaggerated fear responses to cues associated with the trauma and difficulty suppressing fear behavior even when these cues no longer predict danger [19], [20]. In rats, this behavioral phenotype can be modeled by producing elevated startle response to acoustic stimuli and impaired fear extinction. Rats, like humans, show heterogeneity in post-trauma anxiety responses and have been previously classified as those with a PTSD-like phenotype based on their lasting elevation of post-trauma acoustic startle responses (ASR) and anxiety-like behavior in the elevated plus maze (EPM). Encouragingly, the percentage of rats identified with this combination of criteria was 20–25%, similar to the incidence rate of PTSD in humans [21], [6].

Attempts at pre-trauma classification, however, have yielded limited success. Previous studies have shown no relationship between behavior during a traumatic event and impaired extinction [22]; additionally, it is not known if pre-classification based on ASR alone will predict impaired extinction, although it can predict elevated startle [23]. One interpretation of such findings is that there is no identifiable population predisposed to impaired extinction and elevated startle, but, rather, these develop solely as a consequence of the traumatic event. We tested an alternative hypothesis that predispositions do exist and they can be identified prior to the emotional trauma. Specifically, we tested whether predispositions to a more comprehensive PTSD-like behavioral phenotype which includes elevated ASR and impaired fear extinction, could be predicted before the trauma, based on ASR and EPM measures. The results only partially support this hypothesis: pre-classification is possible, but only after the animals have experienced a mild stressor, which by itself does not induce the PTSD-like phenotype. Our investigations also compare what aspects of post-trauma behaviors can be predicted based on either of the two classification factors alone (ASR and EPM measures).

## Results

### Behavioral screening of rats before a traumatic event can predict impaired extinction of fear behavior and lasting elevated startle

**Figure 1:**
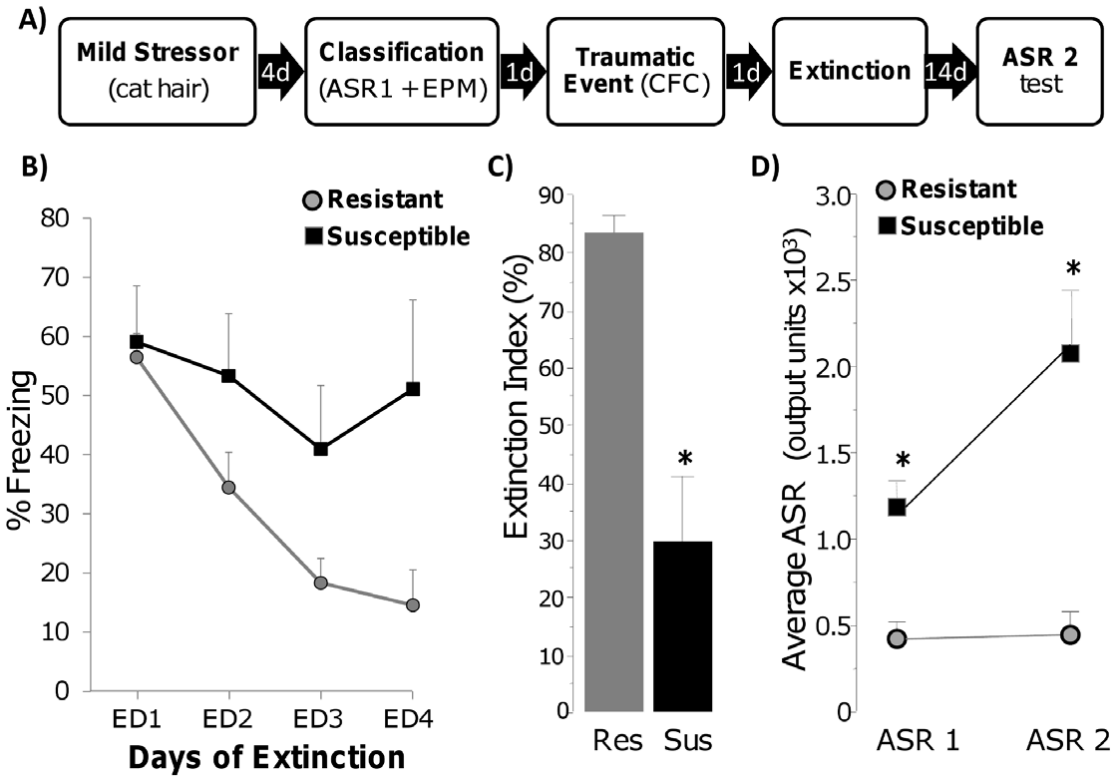
Susceptible rats show impaired rate and magnitude of extinction and sustained elevation in acoustic startle response after a traumatic event. A) Experimental design: ASR = acoustic startle response; EPM = elevated plus maze; CFC = contextual fear conditioning. B) Freezing during daily extinction sessions of Resistant (gray circles) and Susceptible (black squares) rats. C) Magnitude of extinction in Resistant (Res) and Susceptible (Sus) rats; * p<0.01. D) Acoustic startle response at classification (ASR 1) and 3 weeks post trauma (ASR 2); * p<0.001.

We tested the hypothesis that impaired extinction and elevated startle response after an emotionally traumatic event can be predicted based on anxiety-like behavior in the elevated plus maze (EPM) and acoustic startle responses (ASR) before the event. The presented results were derived from three replications of this experiment. As illustrated in Fig. 1A, four days after exposure to a mild stressor (cat hair), animals (n = 51) were tested for ASR and anxiety-like behavior in the EPM and classified based on a set of criteria determined *a priori* that were derived based on pilot experiments (see Methods). Four days were allowed to ensure that the classification was not based on the initial stress response to the cat hair. *Post-hoc* analysis of the behavior of the rats in the presence of the cat hair revealed that those classified as Resistant (n = 13) and Susceptible (n = 9) had a similar aversive response to the cat hair: they showed a similar degree of freezing and number of contacts (F(1, 20) = 1.2 and 0.06, respectively, p>0.2).

The emotionally traumatic event, contextual fear conditioning (CFC), induced robust fear in most rats, as evidenced by notable freezing behavior during the training (mean/SE = 46.0/7.4 for Resistant rats and 47.7/5.5 for Susceptible rats). Three rats (2 Resistant and 1 Susceptible) were excluded from further analyses as they did not meet the training criterion (see Methods). Both groups acquired fear of the context to the same degree, as there was no group difference in freezing behavior during the training (F(1, 17) = 0.02, p = 0.87).

Both groups could retrieve and express the CFC memory to the same degree, as evidenced by a similarly high degree of freezing behavior when the rats were tested the next day in the same context without foot shock (Fig. 1B, Extinction day 1 (ED1)). However, Resistant rats quickly learned to suppress freezing behavior when repeatedly exposed to the same context, while Susceptible rats did not. There was a significant extinction effect (F(3, 51) = 28.97, p<0.0001) and group×extinction interaction (F(3, 51) = 7.05, p<0.001) indicating differences in the rate of extinction. In addition to differences in the rate of extinction, we examined differences between Susceptible and Resistant rats in the magnitude of extinction by assessing an Extinction Index, which is the percent reduction in freezing from ED1 to ED4. While Resistant rats showed a large magnitude of extinction, Susceptible rats did not (F(1, 17) = 10.33, p<0.01, Fig. 1C).

Susceptible rats also had lasting elevated startle responses after the traumatic event compared to Resistant rats (Fig. 1D). The ASR of Resistant (n = 10) and Susceptible (n = 6) rats from two of the three experimental replications was measured 3 weeks after CFC (ASR 2). Susceptible rats had higher ASR 2 than Resistant rats (group effect F(1, 14) = 30.20, p<0.0001). Importantly, while the ASR of Resistant rats remained non-elevated from ASR 1 to ASR 2 testing, ASR 2 of Susceptible rats was elevated above that of their ASR 1 levels (ASR effect F(1,14) = 16.33, p<0.01, group×ASR interaction (F(1,14) = 14.66, p<0.01, and p<0.001 for Susceptible vs. Resistant at ASR 2 testing).

### Lasting elevated startle responses in Susceptible rats is specific to having experienced a traumatic event

**Figure 2:**
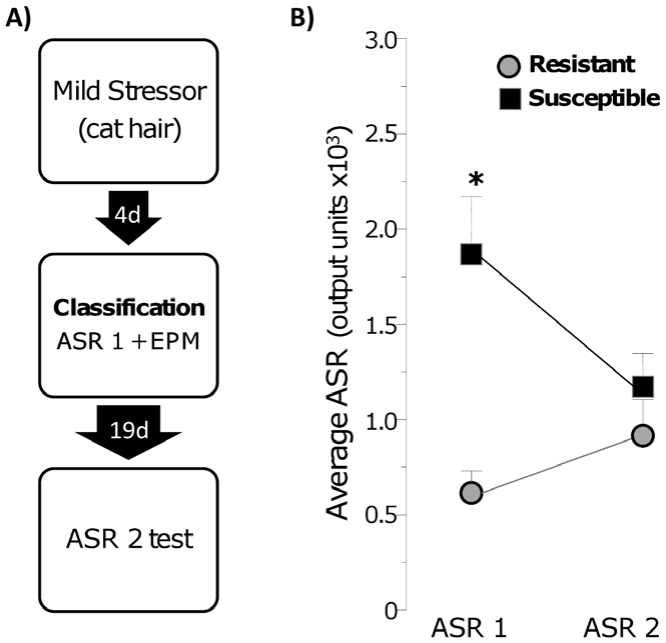
Exposure to a mild stressor does not induce lasting elevation in acoustic startle responses. A) Experimental Design B) Acoustic startle response at classification (ASR 1) and 3 weeks post trauma (ASR 2); * p<0.001.

The observed lasting elevation in ASR in Susceptible rats may result from the exposure to the mild stressor, rather than a specific consequence of the traumatic event (CFC). This hypothesis was tested in a different group of rats that were subject to the mild stressor, classified with the ASR/EPM criteria and then tested for ASR 3 weeks later (Fig. 2A). Naturally, Susceptible rats had higher ASR than Resistant rats during the classification testing, because ASR 1 was a classification criterion (ASR 1, p<0.001, with overall group effect F(1, 11) = 10.77, p<0.01). However, they had an ASR similar to that of Resistant rats during testing 3 weeks later (ASR 2) (ASR factor: F(1, 11) = 0.29, p = 0.59 and significant group×ASR interaction: F(1, 11) = 5.25, p<0.05, Fig. 2B). Therefore, elevated startle after cat hair exposure can be detected in susceptible rats at 4 days after exposure, but this elevation is no longer seen at 3 weeks. Importantly, these data show that the elevated startle observed at 3 weeks after CFC is specific to the traumatic experience and is not induced by the mild stressor.

### Brief exposure to cat hair is a mild stressor

**Figure 3:**
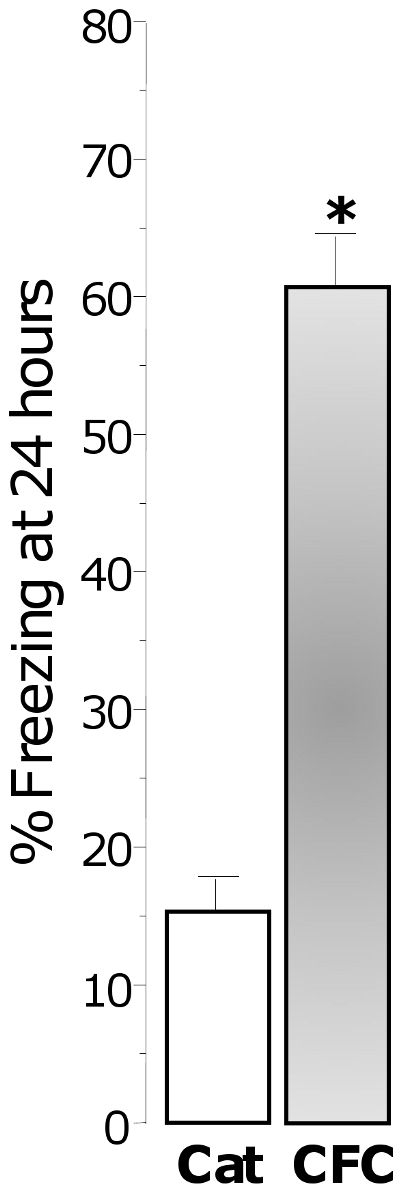
Brief exposure to cat hair is a mild stressor that induces significantly lower conditioned freezing than fear conditioning. Freezing measured 24 hours after training in the cat hair (Cat) and fear conditioning (CFC) context.

As previously reported [12], a short exposure to cat hair elicited a range of fear behaviors, including withdrawal to one corner and freezing (15%). However, conditioned freezing in the cat hair context alone 24 hours after the exposure was 4 times less than the freezing observed at 24 hours after footshock (16% vs. 61%, F(1,29) = 44.74, p<0.0001, Fig. 3). Therefore, a brief exposure to cat hair is a mild stressor and not a severely traumatic event comparable to footshock-induced CFC.

### A mild stressor is needed to reveal susceptibility to impaired extinction and elevated acoustic startle

**Figure 4:**
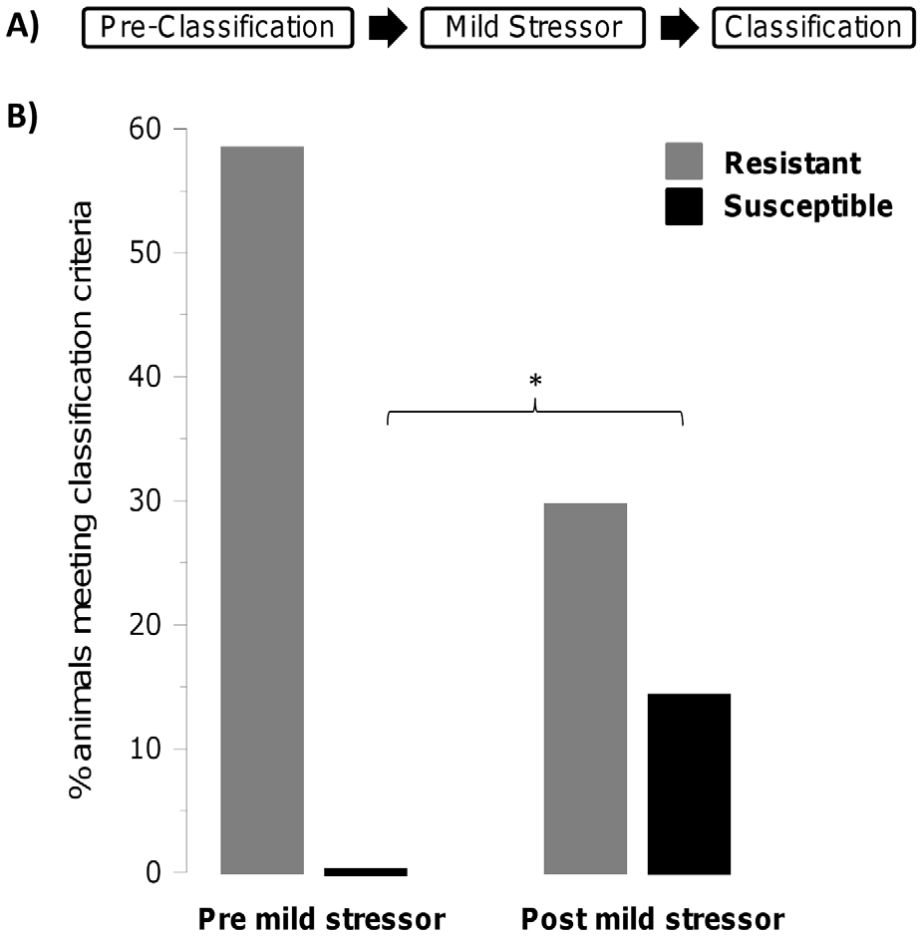
A mild stressor is required to reveal susceptibility to developing impaired extinction and elevated startle. A) Experimental Design: the mild stressor was a brief exposure to cat hair. Classification included EPM and ASR responses. B) Percentage of animals that meet susceptibility and resistance criteria at pre-classification (before cat hair exposure) and at post cat hair classification.

The main finding of the current research is that impaired extinction and prolonged elevated startle can be predicted based on a combination of anxiety and startle responses that are measured after exposure to a mild stressor, but prior to exposure to an emotionally traumatic event. An obvious question arises: can such predispositions be detected at baseline or is a mild stressor needed to reveal them? To answer this question, we first screened rats with the ASR/EPM classification then, after exposing them to the mild stressor, we conducted a second screening using the same criteria (Fig. 4A). Figure 4B shows that while only 1% (1 of 71 rats) met the Susceptible criteria before the cat hair exposure, this percentage increased to 14% (10 of 71) after cat hair exposure. This is just a little lower than the overall rate of susceptibility (17%) seen across all replications (total of 184 rats, including rats from pilot data not reported here that was used to develop the criteria). Conversely, the percentage of rats meeting the Resistant criteria dropped from 59% during the pre-cat hair screening to 30% during the post-cat hair screening. Therefore, a mild stressor is necessary to reveal susceptibility to impaired extinction and elevated acoustic startle.

### Immediate fear response to the mild stressor or to the emotional trauma does not reveal susceptibility to impaired extinction and elevated acoustic startle after the emotional trauma

Exposure to the cat hair stimulus was necessary to reveal predisposition, but could the fear response to this mild stressor predict impaired extinction and elevated ASR after a traumatic event? All 40 rats that met the training criterion were classified as High and Low fear based on whether or not they showed freezing during the cat hair exposure. Animals from both groups acquired fear conditioning at the same rate and to the same degree (group effect: F(1,38) = 2.73, p = 0.11; training effect: F(2,76) = 41.72, p<0.001; no interaction: F(2,76) = 0.29, p = 0.75, data not shown). Similarly, there were no group differences in the rate of extinction (group effect: F(1,38) = 0.57, p = 0.45; training effect: F(3,114) = 35.91, p<0.001; no interaction: F(3,114) = 0.11, p = 0.95), or the magnitude of extinction (F(1,38) = 0.53, p = 0.47). Additionally, there were no group differences for ASR 1 and ASR 2 for the subset of 30 rats that were tested at both time points (group effect: F(1,28) = 1.50, p = 0.23; ASR effect: 1,28) = 4.00, p = 0.06; no interaction: F(1,28) = 0.24, p = 0.62). Further analyses revealed that the percent time spent freezing in the presence of the cat hair was not correlated to either the magnitude of extinction, or the ASR 2 (r^2^<0.001 for both).

The freezing behavior during CFC of the same rats was not correlated to either the magnitude of extinction or to their ASR 2 (r^2^<0.01 for both). These results show that freezing behavior either during the mild stressor or during the emotionally traumatic event cannot be used as a predictor of how well rats will recover from the emotional trauma.

### Does anxiety-like behavior in the elevated plus maze by itself predict impaired extinction and elevated ASR?

**Figure 5:**
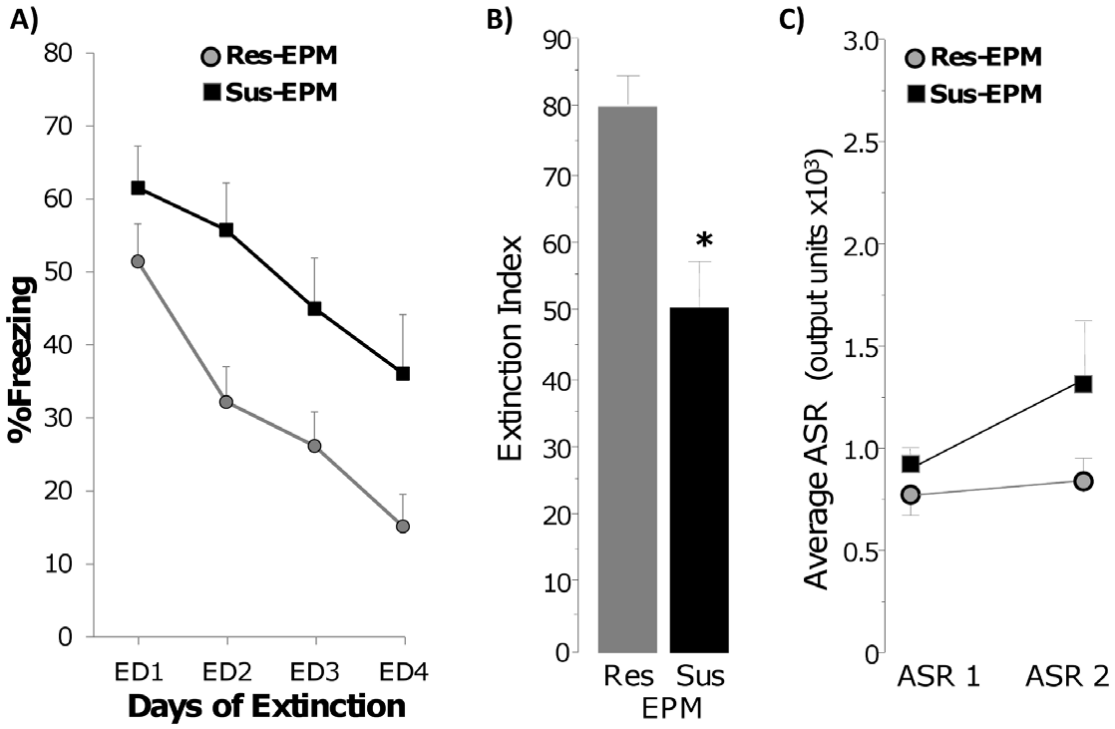
Classification based on post cat hair behavior in the Elevated Plus Maze alone can predict higher levels of freezing and lower magnitude of extinction, but cannot reliably predict impaired rate of extinction and elevated ASR. A) Freezing during daily extinction sessions of rats classified as resistant and susceptible based on the EPM criterion alone (Res-EPM and Sus-EPM, respectively). B) Magnitude of extinction of Res-EPM and Sus-EPM rats; * p<0.01. C) Acoustic startle response at classification (ASR 1) and 3 weeks post trauma (ASR 2). EPM = Elevated Plus Maze.

Although the combined criteria of elevated startle and elevated anxiety in the EPM after exposure to a mild stressor can predict impaired extinction and long-lasting elevated ASR, it is informative to determine whether either criterion alone has the same predictive power. All 40 rats that met the training criterion were classified as susceptible or resistant based on their responses in the EPM alone: Sus-EPM, n = 21 or 53%, and Res-EPM, n = 19 or 48%. There was no group difference in freezing during CFC training (F(1, 38) = 0.03, p = 0.85, data not shown). Fear extinction and startle responses 3 weeks after CFC (ASR 2) are shown in Figure 5. ASR 2 was evaluated in 2 of the 3 replications (n = 30, Sus-EPM = 13 and Res-EPM = 17). Sus-EPM rats had higher levels of freezing throughout the extinction training, as shown by a significant group effect (F(1, 38) = 14.05, p<0.001, Fig. 5A). Sus-EPM rats also showed a lower magnitude of extinction (F(1, 38) = 9.69, p<0.01) and a tendency towards higher ASR 2 (p = 0.061, also a significant group×ASR interaction: F(1,28) = 5.71, p<0.05, Fig. 5C). However, there was no difference in the rate of extinction, no group×extinction interaction (F(3, 114) = 1.53, p = 0.21), and both groups had high magnitude of extinction (>50%, Fig. 5B). Thus, a classification based on EPM alone can predict elevated levels of freezing, but cannot reliably predict elevated ASR and impaired rate of extinction.

### Does elevated ASR after a mild stressor by itself predict impaired extinction and lasting elevation in startle?

**Figure 6:**
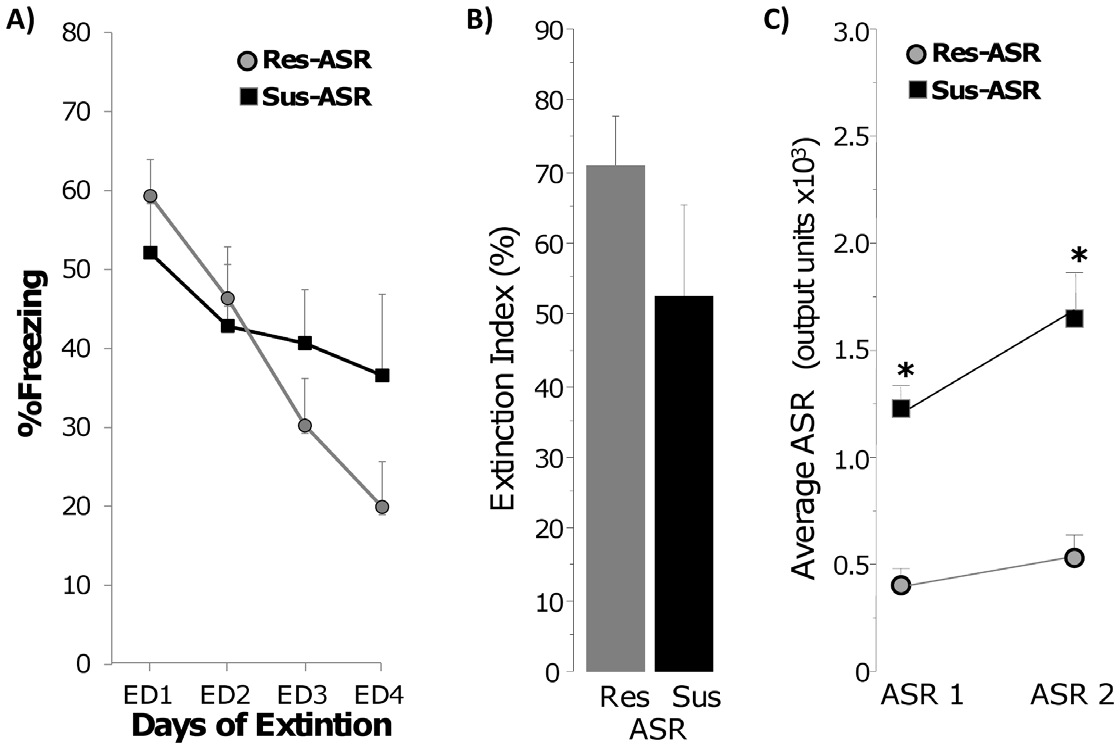
Classification based on post cat Acoustic Startle Response alone predicts post trauma sustained elevation in acoustic startle but not impaired extinction. A) Freezing during daily extinction sessions of rats classified as resistant and susceptible based on the ASR criterion alone (Res-ASR and Sus-ASR, respectively). B) Magnitude of extinction of Res-ASR and Sus-ASR rats. C) Acoustic startle responses at classification (ASR 1) and 3 weeks post trauma (ASR 2); * p<0.001. ASR = Acoustic Startle Response.

In this analysis, extinction and ASR 2 performance of the same 40 rats was evaluated after they were classified *post hoc* based on the startle criterion alone (ASR 1): Sus-ASR, n = 16 or 40%, Resistant-ASR, n = 21 or 53% (there were three Intermediate rats that were excluded from further analyses). There was no group difference in freezing during CFC training (F(1,35) = 0.001, p = 0.97, data not shown). Sus-ASR rats showed an impaired rate of extinction (group×extinction interaction (F(3,105) = 5.24, p<0.01), but showed no difference in the magnitude of extinction (no group effect on the Extinction Index F(1,35) = 1.50, p>0.2), Fig. 6A&B. Both groups also showed high (>50%) overall magnitude of extinction. Not surprisingly, Sus-ASR rats had higher ASR at the time of classification (ASR 1, which is the classification criterion for this set of analyses) and continued to maintain elevated ASR responses 3 weeks after CFC (overall group effect: F(1,28) = 37.19, p<0.001, a significant group difference for ASR 2, p<0.001, and no group×ASR interaction: F(1,28) = 1.22, p = 0.28). Combined with the data from the EPM-alone classification, these data show that a reliable prediction of impaired fear extinction and lasting elevation in the ASR can only be achieved by combing the EPM and ASR criteria, but not by using either criterion alone.

## Discussion

The main finding of the current research is that impaired extinction of conditioned fear and lasting elevated startle responses to loud acoustic stimuli (ASR) can be predicted before exposure to the traumatic event that produces conditioned fear. Thus this model, designed to have predictive power, also has face validity. A second important finding is that this predisposition to a behavioral PTSD-like phenotype is revealed only after experiencing a mild stressor which, by itself, does not induce conditioned fear. We report that susceptibility to develop a PTSD-like phenotype can be predicted by applying the combined criteria of elevated ASR and anxiety-like behavior in the elevated plus maze (EPM) after a pre-trauma exposure to a mild stressor (simulated predator exposure using cat hair). Using either criterion alone can predict different aspects of the post-trauma behavior. Rats classified as susceptible based on their post-cat hair exposure behavior in the EPM (Sus-EPM) show higher levels of conditioned fear a day after fear conditioning and an overall lower magnitude of extinction, compared to Res-EPM rats, but they show no deficits in the rate of fear extinction or the magnitude of ASR measured 3 weeks after emotional trauma. Rats classified as susceptible based on their post-cat hair exposure ASR (Sus-ASR) show the same initial level of fear and similar magnitude of extinction, but they have impaired rate of extinction and higher ASR 3 weeks after emotional trauma. Thus, in order to predict post-trauma susceptibility to both elevated ASR and impaired rate and magnitude of extinction, both ASR and EPM criteria must be applied. The increased predictive power comes at a cost: fewer rats are classified as susceptible (∼18%). However, this percentage is similar to that observed in the human population exposed to traumatic events [1].

It is important to note that freezing during the mild stressor or during the traumatic event was not predictive of how successfully they acquired extinction to the traumatic event or whether or not they developed exaggerated acoustic startle responses. This is consistent with evidence showing that degree of freezing during fear conditioning may predict initial conditioned freezing response, but it does not predict impaired extinction [22]. The finding that classification based on ASR alone can predict lasting elevations in startle almost a month after the traumatic event is consistent with a previous report [23]. On the other hand, pre-classification based on EPM responses alone is sufficient to predict enhanced conditioned fear, but not elevated ASR. These two sets of findings suggest that a pre-trauma test using a reflex or choice measure can predict impairment in the respective modality, but not a combination of both. This is remarkable because there was a higher statistical power in the analyses with either criterion alone, as the group sizes were much larger. Therefore, a combined ASR/EPM measure which includes both reflexive and choice components is best suited for predicting susceptibility to a PTSD-like phenotype in rats.

A surprising finding was that pre-exposure to a mild stressor was required to reveal susceptibility: when rats were classified without first exposing them to cat hair, only 1 of 71 animals met the susceptibility criteria, compared to 10 after such exposure (Figure 4). It should be stressed that the increased percentage of animals that were classified as Susceptible was not due to their immediate response to the mild stressor, because the classification was performed on the 4^th^ day after cat hair exposure when the initial stress response to the cat hair should have subsided. Consistent with this assertion is the fact that 24 hours after the cat hair exposure, rats did not show conditioned freezing (Figure 3). Additionally, *post hoc* analyses of the behavior during the cat hair exposure of Susceptible and Resistant rats did not reveal any group differences.

A criticism may be raised that the observed phenotype results from the exposure to the mild stressor, rather than the traumatic event. This is not the case; the parameters used during the cat hair exposure do not produce contextual fear conditioning (Figure 2) [24]. Furthermore, there was no elevation in the startle response of Susceptible, compared to Resistant, rats 3 weeks after the cat hair exposure (Figure 2), while Susceptible rats showed prominently elevated startle responses at the same time point after fear conditioning (Figure 1). These findings complement existing data on exposure of rats to natural predators and their odor and illustrate that while such exposure is stressful, it has a dose-response effect. Exposure to lower intensity stimuli for a shorter duration, i.e. cat hair for up to 5 min, does not produce fear conditioning and lasting elevation of ASR, while multiple or longer exposure(s), or exposure to a real cat does [25]–[29]. It is this ‘dose-response’ effect of predator/predator odor that has made intense exposure (high-dose) a desirable animal model of PTSD [6], [7], [9], [10], [25]–[27].

Determining whether or not it was necessary to expose rats to a mild stressor prior to classification required assessing anxiety-like behavior in the EPM twice. We were concerned that such repeated testing could bias the EPM data towards classifying more rats as Susceptible (Figure 4), independent of the exposure to the mild stressor, because there is evidence that the time and number of entries in the open arms during repeated testing in the EPM decreases during the second test [30]–[32], although see [32]–[34]. Importantly, under our testing conditions, even when rats were not tested twice in the EPM the percentage of rats classified as Susceptible was similarly high (∼18%, Figure 1) which shows that repeated testing in the EPM cannot account for the higher percent of rats classified as susceptible following the exposure to the mild stressor.

The ability to pre-classify subjects that are likely to develop a PTSD-like phenotype can be essential in helping animal research translate into human studies and real-world treatments. Pre-classification of individuals and populations can aid in selecting the appropriate target population for testing the effectiveness of behavioral and pharmaceutical interventions given either before or shortly after a traumatic event and eventually allow appropriate interventions to be targeted to susceptible patients who are most likely to benefit from treatment. Attempts at pre-trauma classification in both humans and animals are already underway; our current results suggest that adding nonverbal tests can augment the predictive value of verbal self-assessment reports which have shown some efficacy in humans [5].

Lastly, although the ability to predict impaired extinction and lasting elevation in acoustic startle responses has obvious implications for PTSD-related research, it can be a valuable tool for investigating the etiology and pathophysiology of other psychiatric disorders. Such impairments are not unique to PTSD; for example, impaired fear extinction is also common in depression and schizophrenia [35]. A valuable contribution of the presented model is that it provides insights into which criteria need to be used to predict different aspects of impaired extinction and elevated startle.

## Materials and Methods

### Subjects

Young adult (250–300 g) male Sprague-Dawley rats (Charles River Laboratories Inc, MA) were housed in pairs on a 12 hr light/dark cycle (lights on at 7:00 am) with food and water freely available.

### Behavioral procedures

All testing was performed between 9:00 am and 5:00 pm by trained observers blinded to the group assignment of the rats. All behavior, except in the startle chambers, was recorded via an overhead camera. All procedures were approved by the Institutional Animal Care and Use Committee (IACUC), Georgia Health Sciences University, protocol# 08-09-104.

#### Mild stressor

A ball of cat hair, 10 cm in diameter, obtained from a male cat, was placed in one corner of a 35 cm×26 cm×50 cm box. The box was divided into four equal quadrants. Each animal was introduced into the quadrant furthest from the cat hair and allowed to explore the box freely for 3 min. The box was wiped clean between animals. ***Contact*** was scored when the animal's nose was within 2 cm of the cat hair ball. ***Freezing*** was scored when the animal showed no movement except for respiration.

#### Acoustic Startle

Testing was performed in sound attenuated startle chambers (SR-LAB, San Diego Instruments, San Diego, CA) with clear acrylic restraining tubes and background noise of 68 dB. Each animal was presented with fifteen 120 dB acoustic bursts (40 ms each), at random intervals (30–45 s). Acoustic startle response (ASR) was measured as the displacement of the restraining tube detected by a piezoelectric device at its base and reported in output units.

#### Elevated Plus Maze (EPM)

The maze was plus-shaped with four identical 50 cm×10 cm arms, elevated 70 cm above the floor. Two opposite arms were surrounded on three sides by 30 cm tall opaque walls and the other two arms were open, except for a 1 cm high ledge, and dimly illuminated (2 lux). Each animal was introduced in the center area (10 cm×10 cm) facing an open arm and allowed to explore freely for 5 min. Number of arm entries and time spent in each arm were scored. An arm entry was scored when all four paws of the animals entered an arm and time in arm was counted only if all four paws of the animal were within the arm. Two different rooms were used when rats were evaluated in the EPM twice, to make the two exposures as different as possible.

#### Screening Criteria

The animals were classified as Susceptible or Resistant, based on their ASR and EPM scores four days after the mild stressor using *a priori* set criteria as follows:

**Susceptible-** when behavior meets both of the following criteria:

1. Average ASR and 6 or more individual ASR were greater than the group average ASR.
2. No entries into the open arms

**Resistant-** when behavior meets both of the following criteria:

1. Average ASR and more than 7 individual ASR were smaller than the group average ASR.
2. At least 1 entry into the open arms

Animals meeting neither set of criteria were excluded.

#### Traumatic event

Contextual fear conditioning (CFC) was performed in a 50 cm×10 cm×19 cm box. After a three minute habituation period, two shocks (0.7 mA AC, 1000 ms, 30 s apart) were administered as footshocks via stainless steel floor plates electrified by a constant current shock generator. Fear behavior was measured as time spent freezing in the 3 minutes following the second footshock. To ensure that the CFC was indeed a traumatic fear-eliciting event, we set a training criterion of freezing >15% of the post-shock time. The 15% cutoff is based on a meta analysis of the behavioral data from our laboratory acquired by using the same apparatus which showed that <15% freezing at training does not produce reliable fear conditioning, as measured by freezing on the following day.

#### Extinction

Fear extinction to the CFC context was performed by reintroducing the animal into the CFC context for 5 min per day for 4 consecutive days, without footshocks. Freezing was scored. The Extinction Index was a measure of magnitude of extinction and was calculated as: 100-100*(ED4/ED1), where ED1 and ED4 are the percent time spent freezing on extinction days 1 and 4, respectively.

#### Statistical Analyses

Comparisons between groups (Susceptible vs. Resistant) were performed using a single factor ANOVA test (StatView software). A mixed design with repeated measures ANOVA was used when evaluating repeated startle testing, as well as when evaluating group differences and rate of extinction during daily extinction training. When significant overall factor or interaction effects in the RM-ANOVA were observed, a comparison between Susceptible and Resistant rats was done with a t-test. P values smaller than 0.05 were considered statistically significant.
